# Effect of Channel Shape on Performance of Printed Indium Gallium Zinc Oxide Thin-Film Transistors

**DOI:** 10.3390/mi14112121

**Published:** 2023-11-18

**Authors:** Xingzhen Yan, Bo Li, Yiqiang Zhang, Yanjie Wang, Chao Wang, Yaodan Chi, Xiaotian Yang

**Affiliations:** Key Laboratory of Architectural Cold Climate Energy Management, Ministry of Education, Jilin Jianzhu University, 5088 Xincheng Street, Changchun 130118, China; bol222121@gmail.com (B.L.); yqzhang202110@163.com (Y.Z.); wangyanjie@jlju.edu.cn (Y.W.); wangchao@jlju.edu.cn (C.W.); chiyaodan@jlju.edu.cn (Y.C.)

**Keywords:** thin film transistor, ink-jet printing, channel shape, indium gallium zinc oxide

## Abstract

Printing technology will improve the complexity and material waste of traditional deposition and lithography processes in device fabrication. In particular, the printing process can effectively control the functional layer stacking and channel shape in thin-film transistor (TFT) devices. We prepared the patterning indium gallium zinc oxide (IGZO) semiconductor layer with Ga, In, and Zn molar ratios of 1:2:7 on Si/SiO_2_ substrates. And the patterning source and drain electrodes were printed on the surface of semiconductor layers to construct a TFT device with the top contact and bottom gate structures. To overcome the problem of uniform distribution of applied voltages between electrode centers and edges, we investigated whether the circular arc channel could improve the carrier regulation ability under the field effect in printed TFTs compared with a traditional structure of rectangular symmetry and a rectangular groove channel. The drain current value of the IGZO TFT with a circular arc channel pattern was significantly enhanced compared to that of a TFT with rectangular symmetric source/drain electrodes under the corresponding drain–source voltage and gate voltage. The field effect properties of the device were obviously improved by introducing the arc-shaped channel structure.

## 1. Introduction

With the rapid development of electronic equipment, the demand for simplification of the preparation process of display drive units is also gradually increasing. Among them, the construction of thin-film transistors (TFTs), as the core technology of flat panel displays, has been a hot research field [1,2,3]. High-performance TFTs have proved to be one of the most challenging components in functional integrated circuits [4,5,6]. In terms of device preparation, the construction of TFT devices mainly relies on traditional vacuum deposition technology and lithography treatment, but it will be limited by the process environment and complicated process [7,8]. As micro-printing technology matures, printed TFT devices have become a new and transformative industry technology in the field of display manufacturing. The technology of solution-processed TFT devices has rapidly developed in terms of new materials, process optimization, and novel synthesis for high performance [9,10]. The process of printing TFTs can be further simplified by directly realizing the controllable patterning of film on substrates, which has become a new research hotspot in this field [11,12].

For material selection, several types of semiconductors have potential for use in large-size, low-cost, and low-power TFT devices with printing methods, such as metal oxide materials [13,14], organic semiconductors [15,16], and carbon nanotubes [17,18]. In contrast, inorganic oxides have appealing properties, such as environmental and electrical stability, element ratio adjustability, and transparency, which are required for printable driver devices [14,19]. Printed TFTs based on metal oxide semiconductors, such as indium oxide [20,21], zinc tin oxide [22], indium zinc tin oxide [8], indium tin oxide [23,24], and indium gallium zinc oxide (IGZO) [25,26], can obtain good field effect performance and become the focus of research. Among them, amorphous IGZO materials have been reported to have advantages in mobility and stability that can be applied to TFTs [27]. Furthermore, indium as a doping cation affects the electronic configuration in channel layers, and the stability of the gallium–oxygen bond suppresses the generation of oxygen vacancies, thus decreasing the free electron concentration [28].

For structural design, printed TFTs also showed advantages in the fabrication of multilayer structural stacking, including the patterning design of semiconductor layers and the accurate coverage of patterned source/drain electrodes [29]. Therefore, the circular arc electrode structure can be explored to break the traditional rectangular symmetry structure by printing technology in TFT devices. The structure of a circular arc can solve the difference in electric field distribution between electrode centers and edges compared with a traditional structure of rectangular symmetry [30]. In this paper, we used the printing process to construct the IGZO TFTs with a bottom gate consisting of n-type doped silicon (Si) wafers and a top contact consisting of silver nanoparticles. We propose a circular arc source/drain electrode structure to improve the field effect performance of printed TFTs. We investigated the arc channel to effectively improve the uniformity of potential distribution in the IGZO channel and enhance the electrical properties of the TFT devices.

## 2. Materials and Methods

### 2.1. Preparation of TFT Devices

**Figure 1:**
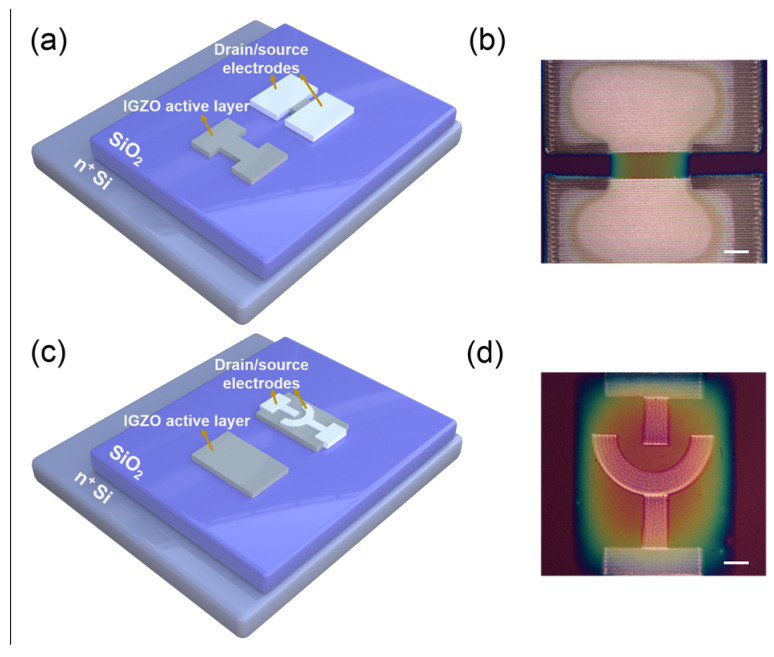
Schematic illustrations of the procedures for fabricating printed IGZO TFTs with the symmetrical rectangular source/drain electrodes (**a**) and circular arc source/drain electrodes (**c**). Microscope images of the rectangular (**b**) and circular arc channel patterns (**d**) of IGZO/SiO_2_/Si TFTs. The scale bar is 100 μm.

An n-type phosphorus-doped Si wafer and a silicon dioxide (SiO_2_) layer with a thickness of ~285 nm from HEFEI KEJING Materials Tech Co., Ltd. (Hefei, China) were used as the bottom electrode and insulation layer of TFT devices. The colloidal precursors were prepared from gallium nitrate [Ga(NO_3_)_3_], indium nitrate [In(NO_3_)_3_], and zinc acetate [Zn(CH_3_COO)_2_] purchased by Shanghai Aladdin Biochemical Technology Co., Ltd. Moreover, they were dissolved in 5 mL of methyl glycol to achieve 0.4 M of IGZO solution with Ga, In, and Zn molar ratios of 1:2:7. Furthermore, 1.2 mL of ethanolamine and 300 µL of glacial acetic acid were successively added to the IGZO solution to act as stabilizers. The Si/SiO_2_ substrates were ultrasonic cleaned in acetone, alcohol, and DI water for 10 min, respectively. The treated substrates were dried with nitrogen and then cleaned with argon plasma. The plasma processing conditions were set at a power supply of 75 W and a processing time of 10 s. Removing the impurities adsorbed on the substrate can enhance the wetting of the IGZO solution onto the surface of Si/SiO_2_ substrates. This allows for more accurate printing of the patterning semiconductor layer on the substrate by a Sonoplot printing instrument. However, there will still be coffee rings on the semiconductor layer, which is an important problem that many research groups need to solve in printed devices. The coffee ring effect originates from the significant outward flow of dropped inks, and any roughness in the printed device will have an adverse effect on the electrical performance because of defects formed in subsequently printed layers [31]. In order to avoid the influence of the coffee ring effect on comparability, we constructed the channel in the middle of the semiconductor layers to ensure the uniformity of the field effect performance for the TFTs with different printed channel shapes. A commercial silver nanoparticle ink (SILVER NANOPASTE NPS-J, purchased by Harima Materials Co., Ltd. (Tokyo, Japan) was used as a printed conductor. The patterned source and drain electrodes were prepared on the IGZO semiconductor layer using a SIJ printing instrument with a 150 V printing voltage, 2 mm/s printing speed, 1000 Hz release frequency, and a 30 µm distance between the tip and semiconductor layers. Then, the source/drain electrodes were annealed at 200 °C for 30 min to remove the organics in the silver nanoparticles printing ink. The thickness of the resulting electrodes was about 100 ± 20 nm. The schematic illustration of the structure of printed IGZO TFTs is shown in Figure 1. The channel length/width (L/W) ratio of TFT devices is a geometrical factor in calculating field effect mobility (μ_eff_). The rectangular symmetrical channel was commonly used in TFTs, as shown in Figure 1a. The channel length and width of the IGZO TFT were about 100 μm and 300 μm, respectively. In contrast, the circular arc channel can solve the difference in electric field distribution between electrode centers and edges, as shown in Figure 1b. Moreover, this channel design can reduce the waste of electrode materials and provide more channel contacts for TFT applications in photoelectric detection and gas sensing.

### 2.2. Characterization

The microstructures of patterned channel layers were observed by means of an optical microscope (AxioScope A1, Carl Zeiss, Oberkochen, Germany). Patterned channel layers and source/drain electrodes were, respectively, prepared by inkjet printing instruments (Microplotter II, Sonoplot, Middleton, WI, USA and S050, SIJ, Ibaraki, Japan). Output characteristics and transfer characteristics of TFTs were measured by a semiconductor parametric instrument (B1500A, Keysight, Santa Rosa, CA, USA). The argon plasma was produced by a metal and organic evaporation system (LN-2063, SHENYANG LINING, Shenyang, China).

## 3. Results and Discussion

**Figure 2:**
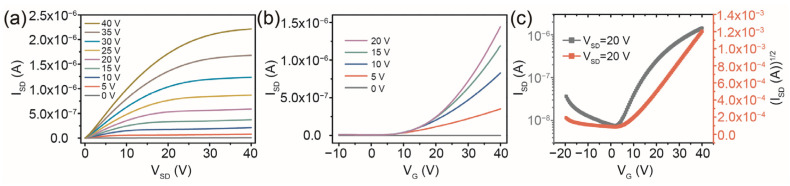
(**a**) Output characteristics of a printed IGZO TFT with symmetrical rectangular source/drain electrodes. (**b**,**c**) Transfer characteristics of the rectangular channel IGZO TFT.

The patterned IGZO semiconductor layers were printed onto the Si/SiO_2_ substrates. In order to prevent cracks in the patterned IGZO, the annealing temperature of the pristine IGZO channel layer was set to 350 °C for 1 h, with a transition annealing step at 450 °C for 1 h, and finally annealing for 1 h at 550 °C in the air atmosphere with a heating rate of 5 °C/min. The thickness of the patterned IGZO semiconductor layer after annealing treatment was about 50 nm. The dumbbell shape of the IGZO semiconductor layers was designed to get more carrier injection when the drain–source voltage (V_SD_) was applied. The printed IGZO was covered with symmetrical rectangular source/drain electrodes to obtain a TFT with a rectangular channel pattern. Figure 2a shows the drain–source current (I_SD_) versus the V_SD_ output characteristics of the IGZO TFT at gate voltages (V_GS_) from 0 to 40 V with the rectangular channel pattern. The curves show typical n-type field effect properties with an obvious transition from linear to saturation behavior in the IGZO channel. Figure 2b,c shows the transfer curves of the printed IGZO TFT with the rectangular channel. The threshold voltage (V_th_) of ~6.1 V was estimated by extrapolating the linear portion of the (I_SD_)^1/2^ versus V_G_ curves at V_SD_ = 20 V in the typical transfer curves. The values of field effect mobility (μ_eff_) and subthreshold swing (SS) were extracted from the transfer characteristics of the devices using the following formula:

(1) $$\mu_{\mathrm{eff}}=\frac{2L}{{\mathrm{WC}}_{i}}{\left(\frac{\partial \sqrt{I_{\mathrm{SD}}}}{\partial V_{G}}\right)}^{2}$$

(2) $$\mathrm{SS}=\frac{\partial V_{G}}{\partial \left(\log_{10}I_{\mathrm{SD}}\right)}$$

where C_i_ is the capacitance of the SiO_2_ gate insulator with a thickness of about 285 nm per unit area, and L and W are the channel length and width of the patterned IGZO, respectively. The printed IGZO TFT with a rectangular channel exhibited a μ_eff_ value of 0.069 cm^2^ V^−1^ s^−1^, a SS of 8.6 V dec^−1^, and an on/off ratio (I_on_/I_off_) of 1.8 × 10^2^.

**Figure 3:**
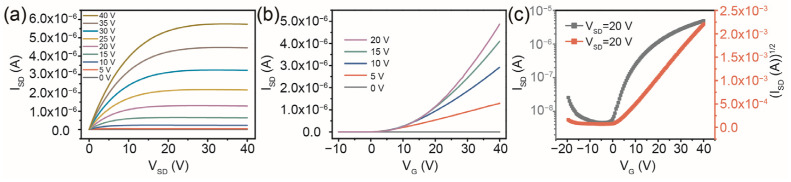
(**a**) Output characteristics of a printed IGZO TFT with the circular arc source/drain electrodes. (**b**,**c**) Transfer characteristics of the circular arc channel IGZO TFT.

In contrast, we presented a structure of circular arc source/drain electrodes, which can solve the difference in electric field distribution between electrode centers and edges compared with a traditional structure of rectangular symmetry (see Figure 1b). The arc channel is more conducive to the effective injection of carriers through the source/drain electrodes. This was demonstrated by the value of the I_SD_ in the output curves of the IGZO TFT with a circular arc channel pattern under a V_G_ value of above 20 V, which was more than twice as large as that of a TFT with the rectangular symmetric source/drain electrodes at the corresponding V_SD_ (see Figure 3a). From the representative transfer curves as shown in Figure 3b,c, the TFT device with a circular arc channel exhibits an n-type behavior with a V_th_ of ~2.2 V, a SS of 4.4 V dec^−1^, and an I_on_/I_off_ of 1.1 × 10^3^ at a V_DS_ of 20 V. The geometrical factor as the effective channel length/width (L/W) of TFT devices with an arc channel can be expressed as follows [32,33]:

(3) $$\frac{L}{W}=\frac{\ln\left(R_{2}/R_{1}\right)}{\pi }$$

where R_1_ is the radius of the internal source electrode (~50 µm) and R_2_ is the sum of the radius of the internal source electrode and the length of the IGZO channel (~50 + 100 µm). By substituting the value of L/W into Equation (1), a μ_eff_ value of 0.208 cm^2^ V^−1^ s^−1^ was obtained for an IGZO TFT with the circular arc source/drain electrodes. The μ_eff_ value of the printed TFT was increased by two times with a circular arc IGZO channel. The value of I_on_/I_off_ increased by about an order of magnitude, which can more effectively reduce noise interference during TFT device operation. The reduction in the value of V_th_ can effectively reduce the power consumption of the printed TFTs. This was due to the fact that the uniformly distributed V_SD_ promoted the efficient injection and migration of the carriers in the channel.

**Figure 4:**
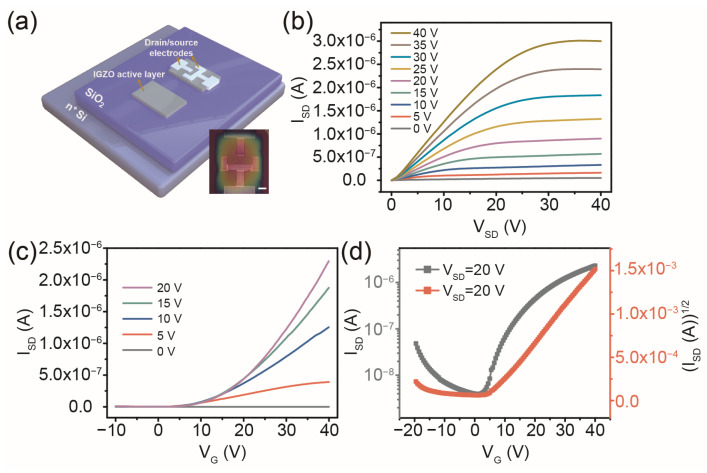
(**a**) Schematic illustration of fabrication of a printed IGZO TFT with a rectangular groove channel. The insert shows a microscope image of an IGZO/SiO_2_/Si TFT with a rectangular channel pattern. The scale bar is 100 μm. (**b**) Output characteristics of the printed IGZO TFT with the rectangular groove channel. (**c**,**d**) Transfer characteristics of the rectangular groove channel IGZO TFT.

In contract, we changed the circular arc channel into a corresponding rectangular groove channel to verify the improvement of the field effect performance of the printed TFT devices (see Figure 4a). As shown in Figure 4b, the transition from the linear to the saturation regime and a good regulating effect on the I_SD_ are observable in the output characteristics of the IGZO TFT with a structure of rectangular groove channels under different voltages. However, under the same V_G_, the value of I_SD_ was smaller than that of the printed TFT with a circular arc channel and larger than that of the device with a rectangular symmetrical channel. This showed that the arc channel was not affected by the electrode tip on the potential distribution, and the plug-in structure of the source/drain electrodes was more conducive to carrier transport. Figure 4c,d illustrates the transfer characteristics of the IGZO TFT fabricated by ink-printing with a rectangular groove channel. The TFT device exhibited ~3.1 V V_th_, 4.9 V dec^−1^ SS, and 5.9 × 10^2^ I_on_/I_off_ at a V_DS_ of 20 V. The channel L/W of this type of transistor was estimated using the following formula [34]:

(4) $$\frac{L}{W}=\frac{L}{a+2b}$$

where L is the length of the channel; a and b are the width of the source electrode (~100 µm) and the distance between the two ends of the groove structure of the drain electrode and the source electrode (~100 + 200 µm), as shown in Figure 4a. By substituting the value of L/W into Equation (1), a μ_eff_ value of 0.093 cm^2^ V^−1^ s^−1^ was obtained for the IGZO TFT with a rectangular groove channel. Compared with TFT with a circular arc channel structure, this type of TFT device still has a certain gap in the performance of carrier regulation, which proves that the symmetry of potential distribution in the channel is helpful in improving the electrical properties of TFT devices. Finally, the field effect parameters of the TFT devices with various printed channel shapes are summarized in Table 1. Compared with the circular arc channel TFT, the other two types of TFT devices still have a certain gap in the performance of carrier regulation, which proves that the symmetry of potential distribution in the channel is helpful in improving the electrical properties of TFT devices. Compared with the mature TFTs, our printed preparation process still has a certain gap in the field effect characteristics, but we will improve the performance of the TFTs by modifying the interface next, especially to improve the problems that silver nanoparticles as electrodes will diffuse into the channel layer with the application of voltage and the defects at the interface between the channel layer and electrodes will affect the carrier transport.

## 4. Conclusions

We investigated a printing method to construct the bottom gate and top contact TFT devices with different IGZO channel patterns. We prepared an IGZO ink for printing with Ga, In, and Zn molar ratios of 1:2:7. A structure of a circle-arc channel was presented to solve the difference in electric field distribution between electrode centers and edges for TFTs. Compared with the traditional rectangular symmetrical and rectangular groove structures of source/drain electrodes for TFTs, the electrical performance of the device was significantly improved by introducing the circle-arc channel. The optimized printed TFT exhibited a V_th_ of ~2.2 V, a SS of ~4.4 V dec^−1^, an I_on_/I_off_ of ~1.1 × 10^3^, and a μ_eff_ value of 0.208 cm^2^ V^−1^ s^−1^. Among them, the V_th_ and μ_eff_ values are greatly increased, which is conducive to reducing the power consumption and improving the carrier regulation ability in printed TFT devices.

## Figures and Tables

**Table 1:** The field effect parameters of the TFTs with various printed channel shapes.

Type	On/Off Ratio	Field Effect Mobility (cm^2^ V^−1^ s^−1^)	Threshold Voltage (V)
Symmetrical rectangular channel	1.8 × 10^2^	0.069	6.1
Rectangular groove channel	5.9 × 10^2^	0.093	3.1
Circular arc channel	1.1 × 10^3^	0.208	2.2

## Data Availability

Data are contained within the article.
